# Myosin Va Plays a Role in Nitrergic Smooth Muscle Relaxation in Gastric Fundus and Corpora Cavernosa of Penis

**DOI:** 10.1371/journal.pone.0086778

**Published:** 2014-02-06

**Authors:** Arun Chaudhury, Vivian Cristofaro, Josephine A. Carew, Raj K. Goyal, Maryrose P. Sullivan

**Affiliations:** 1 Divisions of Surgery, VA Boston Healthcare System and Harvard Medical School, Boston, Massachusetts, United States of America; 2 Division of Urology, VA Boston Healthcare System and Harvard Medical School, Boston, Massachusetts, United States of America; 3 Division of Medicine, VA Boston Healthcare System and Harvard Medical School, Boston, Massachusetts, United States of America; Temple University School of Medicine, United States of America

## Abstract

The intracellular motor protein myosin Va is involved in nitrergic neurotransmission possibly by trafficking of neuronal nitric oxide synthase (nNOS) within the nerve terminals. In this study, we examined the role of myosin Va in the stomach and penis, proto-typical smooth muscle organs in which nitric oxide (NO) mediated relaxation is critical for function. We used confocal microscopy and co-immunoprecipitation of tissue from the gastric fundus (GF) and penile corpus cavernosum (CCP) to localize myosin Va with nNOS and demonstrate their molecular interaction. We utilized *in vitro* mechanical studies to test whether smooth muscle relaxations during nitrergic neuromuscular neurotransmission is altered in DBA (dilute, brown, non-agouti) mice which lack functional myosin Va. Myosin Va was localized in nNOS-positive nerve terminals and was co-immunoprecipitated with nNOS in both GF and CCP. In comparison to C57BL/6J wild type (WT) mice, electrical field stimulation (EFS) of precontracted smooth muscles of GF and CCP from DBA animals showed significant impairment of nitrergic relaxation. An NO donor, Sodium nitroprusside (SNP), caused comparable levels of relaxation in smooth muscles of WT and DBA mice. These normal postjunctional responses to SNP in DBA tissues suggest that impairment of smooth muscle relaxation resulted from inhibition of NO synthesis in prejunctional nerve terminals. Our results suggest that normal physiological processes of relaxation of gastric and cavernosal smooth muscles that facilitate food accommodation and penile erection, respectively, may be disrupted under conditions of myosin Va deficiency, resulting in complications like gastroparesis and erectile dysfunction.

## Introduction

Nitric oxide (NO) produced by splice variants of the enzyme neuronal nitric oxide synthase (nNOS) has been shown to be a major inhibitory neurotransmitter at smooth muscle neuromuscular junctions. Smooth muscle relaxation facilitated by NO released from nerve varicosities subserves essential physiological functions in many organ systems that are as diverse as gastric motility and penile erection [1]–[4]. In these prototypical examples of nitrergic neurotransmission, impaired relaxation has been shown to cause, respectively, a variety of gastrointestinal motility disorders including loss of gastric accommodation [5] and erectile dysfunction [6].

During nitrergic neurotransmission, NO is synthesized *de novo* and released on demand from nitrergic varicosities [7]. Failed nitrergic neurotransmission may result from an absence or critical reduction in the amount of nNOS or impairment of its catalytic function. Many factors determine the catalytic activity of nNOS including dimerization [8]–[9], and its interaction with regulatory proteins and calcium ions [10]–[12]. Moreover, the subcellular localization of nNOS also regulates its catalytic activity [13]–[15]. Within enteric varicosities, membrane localization of nNOSα has been shown to be necessary for optimal NO synthesis [15]. nNOSα has an N-terminal PDZ domain which binds the PDZ domains of PSD95, a membrane bound scaffolding protein concentrated at ‘active zones’ [8], [15]. These zones are characterized by a supra-molecular assembly of nNOSα dimers, close to the source of calcium surge during an action potential, thus optimizing NO synthesis and ensuring focal release of NO [14]–[15].

While nNOS tethering to the membrane has been well studied, the mechanisms involved in the translocation of nNOSα from the cytosol to the varicosity membrane have not been fully clarified. It was reported that PIN (protein inhibitor of nNOS), also identified as LC8 (light chain 8 kDa), may be involved in the transport of cytosolic nNOSα to varicosity membranes [16], and regulation of nNOSα activity [16]–[17]. LC8 has been demonstrated not only in enteric nerve varicosities [16], but also in penile cavernosal nerves [17], central nervous system (CNS) nerve terminals [18] and other cellular sites where nNOS transcytosis has been reported [19]. In the gut, LC8 acts as an adaptor protein that binds nNOSα to myosin Va [20], an unconventional non-muscle cytoskeletal motor protein known to transport a variety of intracellular cargo along actin filaments. A recent study in mice with mutation in the myosin Va gene showed that membrane associated nNOSα is reduced in isolated enteric nerve varicosities and that *in vitro* NO production as well as nitrergic inhibitory junction potentials in the stomach are significantly diminished [20]. These results provided evidence that myosin Va may be involved in intravaricosity translocation of nNOSα to the membrane and hence nitrergic neurotransmission. However, it is not known whether mechanical relaxation of the gastric smooth muscles, which precedes gastric accommodation, is impaired in myosin Va deficient mice.

Analogous to its role in the stomach, nitrergic neurotransmission provokes smooth muscle relaxation in the corpus cavernosum of the penis (CCP). Relaxation of cavernosal smooth muscle permits filling of the CCP with blood leading to penile erection [3]–[6]. The nNOS splice variant expressed in penile nitrergic nerves (PnNOS) bears molecular homology to nNOSµ in striated muscles [21] and differs structurally from the nNOS splice variant expressed in the stomach (nNOSα). Despite these isoform differences, the biology of nNOS appears comparable in the penis and stomach, including interaction of the enzyme with LC8 and its localization to varicosity membranes [17], [20]. However, the role of myosin Va in PnNOS mediated nitrergic relaxation in the penis has not been investigated previously.

The purpose of the present study was to determine the functional role of myosin Va in nitrergic neurotransmission in representative, yet distinct smooth muscle systems. Therefore, nitrergic nerve mediated smooth muscle relaxation was compared in GF and CCP of normal and myosin Va-deficient mice.

## Materials and Methods

The study protocol was conducted according to the recommendations in the Guide for the Care and Use of Laboratory Animals of the NIH and approved by the Institutional Animal Care and Use Committee of VA Boston Healthcare System (#101W).

### DBA Mice

Male DBA mice (DBA/2J strain, Jackson Labs, 27.5±1.8 weeks old) were used as models of myosin Va deficiency. DBA mice lack cellular myosin Va due to an inherited frameshift mutation in the MYO5A gene, induced by a proviral/inactivating insertion [22]. This mouse strain derives its name from its coat-color alleles called dilute (d), brown (b), and non-agouti (a). The lightening of the color coat is caused by defective transport of melanin containing melanosomes in melanocytes due to deficiency of myosin Va [23]. Male C57BL/6J wild type (WT) mice with black coat color served as controls (22.9±2.2 weeks old).

### Confocal Microscopy

Tissue samples from the GF of the stomach and the corpora cavernosa of the penis (CCP) were harvested from WT mice immediately after euthanasia, embedded in cryoprotective compound and quickly frozen in dry ice. Tissue sections were cut on a cryostat (12 µm), fixed in cold acetone (10 minutes), and incubated for 1 hour in blocking solution (PBS 10% donkey serum, 0.3% triton-X). After blocking, sections were first incubated overnight at 4°C with rabbit anti-myosin Va primary antibody (1∶500, Sigma). For detection of primary antibodies from the same host species, tissue sections were incubated with excess Fab fragment goat anti-rabbit IgG (10 µM/ml; 1 hour RT) to present the first antibody as a different species. A fluorescent-conjugated donkey anti-goat secondary antibody (Alexa Fluor 488, Invitrogen) was used to detect the first primary antibody. After extensive washing, the same sections were incubated overnight with anti-rabbit nNOS, (1∶100 Santa Cruz), followed by fluorescent-conjugated secondary antibody (Alexa Fluor donkey anti-rabbit 568) for 1 hour at RT. Control slides were prepared as described above with the omission of primary antibodies. Sections were examined using a laser scanning confocal microscope (Zeiss LSM 710). High resolution images were acquired sequentially from separate channels using Zen Imaging software.

### Immunoprecipitation

The molecular interaction between Myosin Va and nNOS in WT fundus and CCP was determined by co-immunoprecipitation (IP) experiments. The mucosa was removed from the GF by microdissection. Tissues were lysed in IP extraction buffer (Dynabeads Co-immunoprecipitation kit, Invitrogen) supplemented with 100 mM NaCl, 2 mM MgCl2, 1 mM DTT, and protease inhibitors for 15 min at 4°C. After incubation, homogenates were centrifuged at 3000×g for 5 minutes and the supernatant representing the total protein lysate was collected. Protein concentrations were determined using the bicinchoninic acid protein assay (BCA) by measuring the absorbance at 280 nm with a biophotometer (Eppendorf). Anti-nNOS antibody, was covalently immobilized onto the surface of M-270 epoxy magnetic beads (Dynabeads® Antibody Coupling Kit, Invitrogen) by incubating the antibody-bead complex for 24 hrs under agitation at 37°C. Equal amounts of the antibody-coupled beads (1.5 mg per reaction) were added to protein lysates from both WT fundus (1.7 mg/ml) and CCP (3.4 mg/ml) and incubated under agitation for 1 hour at 4°C. After incubation, the beads with immobilized proteins were collected by placing the tubes in a magnetic field (DynaMag™-2, Invitrogen) and the remaining supernatants were stored. Immunoprecipitated complexes were eluted from beads by incubation in EB-buffer (Invitrogen) for 5 minutes at RT and separated from the antibody-coupled beads by magnet. Total lysates (15 µg), purified protein complexes and supernatants from the fundus and CCP were resuspended in sample loading buffer, boiled for 5 minutes, loaded onto a tris-acetate SDS polyacrylamide gel and separated by electrophoresis. Proteins were then transferred to a 0.45 µm pore nitrocellulose membrane (Invitrogen). Non-specific binding was inhibited by incubating membranes in Tris Buffered Saline (TBS) with 5% dry milk for 1 hour. Membranes were incubated overnight at 4°C with primary antibodies for myosin Va or nNOS. Unbound antibody was removed by extensive washing with TBS containing 0.05%Tween 20. Membranes were incubated for 1 hour at RT with horseradish peroxidase-conjugated secondary antibody (Santa Cruz). After washing, the membrane was incubated with chemiluminescence substrate (Western Lightning plus-ECL, Perkin Elmer), and immunoreactive bands were visualized by exposure of membrane to radiographic film (Kodak Biomax).

### Preparation of Smooth Muscle Tissues

After euthanasia induced by CO_2_ asphyxiation, animals were weighed and the stomach and the penis were excised and placed in cold Kreb’s solution (NaCl 120 mM; KCl 5.9 mM; NaHCO_3_ 25 mM; Na_2_H_2_PO_4_ 1.2 mM; MgCl_2_ • 6H_2_O 1.2 mM; CaCl_2_ 2.5 mM; dextrose 11.5 mM). The stomach was opened by a longitudinal incision along the greater curvature, and the gastric mucosa was removed from the fundic portion of the organ by stereoscopic micro-dissection. Four longitudinal smooth muscle strips from the fundus were obtained from each animal. The penis was isolated from the mid-shaft to the base of the crura. After removing the fascia and corpus spongiosum, the septum was cut longitudinally to separate the two corpora cavernosa. Cavernosal smooth muscle was exposed by removing the tunica albuginea. At the end of the experiment, the weight of each tissue from the GF and CCP was recorded after blotting on filter paper.

### Muscle Tension Studies

Fundus and CCP smooth muscle tissues were isolated from DBA and WT mice. Each end of the tissue was tied with silk, and transferred to an organ bath containing Kreb’s solution maintained at 37°C and continuously gassed with carbogen (95%O_2_+5%CO_2_). One end of each strip was attached to a fixed hook, and the other end to a force transducer measuring isometric tension (Grass Technologies). Smooth muscle strips from both GF and CCP were stretched under 0.5 grams of tension and equilibrated for 1 hour. Force measurements were displayed on a strip chart recorder (Gould) and digitally acquired by computer (WinDaq Software). After equilibration, tissue strips from the fundus were contracted by addition of carbachol (CCh, 10 µM) to the organ bath and contractile responses in WT and DBA animals were compared. Contractile responses of muscle strips from WT and DBA mice to high extracellular potassium (KCl 120 mM) were also compared. In the CCP, pre-contraction of smooth muscle was achieved by addition of phenylephrine (PE, 100 µM). Guanethidine (1 µM) was added to inhibit noradrenergic transmission [24].

Nerve-mediated relaxation was elicited in pre-contracted tissues by electrical field stimulation (EFS, 40V, 2–64 Hz, 0.5 ms pulse duration, 10 seconds) delivered by platinum electrodes connected to a stimulator (Harvard Apparatus). In smooth muscle tissues obtained from WT, the effect of EFS-induced relaxation in the GF and CCP was investigated in the presence of NOS inhibitor L-nitro-L-Arginine Methyl Ester (L-NAME, 10 µM). The direct effect of nitric oxide on smooth muscle relaxation was examined in both GF and CCP by exposing pre-contracted tissue to submaximal concentrations of NO donor sodium nitroprusside (SNP, 1 µM).

### Data Analyses and Statistics

The amplitude of contractile and relaxation responses were reported as the difference in tension (expressed in Newton) before and after the stimulus and normalized by the weight of the tissue strip (expressed in grams). EFS induced relaxation responses from the fundus were also expressed as a percent change from the active tension achieved by CCh pre-contraction. Data are reported as mean ± SEM. Differences in means for the contractile or relaxation responses to agonists and EFS before and after L-NAME were analyzed by paired student’s t test. Comparison of contractile or relaxation responses between WT and DBA were analyzed by unpaired t-test followed by Holm-Sidak post-hoc analysis. P<0.05 was considered significant.

## Results

### Expression and Distribution of Myosin Va in Fundus and CCP and its Interaction with nNOS

**Figure 1:**
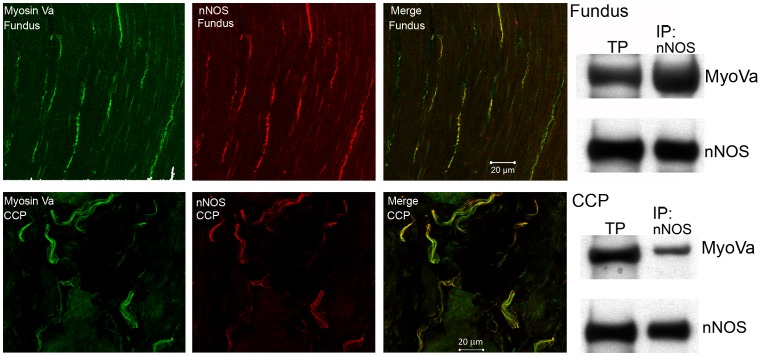
Localization and interaction of myosin Va and nNOS in the fundus and corpora cavernosa (CCP). In both fundus (top panels) and CCP (bottom panels), immunoreactivity for myosin Va (green) is present in nerve fibers that are immunoreactive for nNOS (red). Co-localization (yellow) is present in projected image. Western blot on right shows positive bands for myosin Va and nNOS in total protein lysate (TP) in lane 1. In lane 2, samples were immunoprecipitated for nNOS and blotted for myosin Va to demonstrate molecular interaction between these proteins in fundus (top panel) and CCP (bottom panel).

Myosin Va immunoreactivity was detected in both gastric and penile tissues. Myosin Va staining was specifically localized within nNOS immunoreactive nerve varicosities coursing through smooth muscle bundles in both tissues (**figure 1**). Coarse nerve trunks in the CCP were also positive for myosin Va. Molecular interaction between nNOS and myosin Va, determined by co-immunoprecipitation, was demonstrated in both GF and CCP (**figure 1, right panel** **s**).

### Nerve- mediated Relaxation in Gastric Fundus (GF)

Smooth muscle strips from the fundus of both WT and DBA contracted after the addition of either KCl or CCh. The contractile responses induced by KCl were similar (2.25±0.2N/g and 1.78±1.6N/g respectively in WT and DBA mice, p = 0.07). However, the contractile response to CCh was significantly lower in DBA (2.14±0.18 N/g) compared to WT fundus (3.73±0.3 N/g, p = 0.002). This reduced carbachol response was not further investigated; however, it is possible that it may be due to impaired membrane localization of cholinergic receptors in the smooth muscle since myosin Va has been shown to regulate membrane localization of cholinergic (nicotinic) receptors in striated muscle [25].

**Figure 2:**
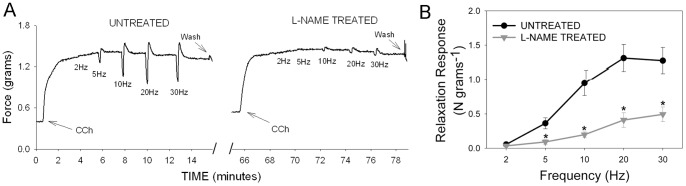
Effect of L-NAME on nerve-mediated relaxation in mouse gastric fundus. (A) Representative tracings before (untreated) and after L-NAME treatment showing pre-contraction induced by carbachol (arrows) followed by relaxation due to increasing frequencies of stimulation in a WT mouse. Note the relaxation response during the stimulation is followed by an off-contraction. (B) Relaxation response to EFS after L-NAME treatment (gray triangles) was significantly lower than baseline relaxation response (black circles). [n = 5; * = significantly lower than baseline response; p<0.0002 by paired t test].

In WT animals, carbachol pre-contracted tissue relaxed in response to EFS in a frequency dependent manner, reaching maximal relaxation at frequencies between 20 and 30 Hz. Administration of L-NAME (10 µM), a non-selective NOS inhibitor, had no effect on the amplitude of CCh-induced pre-contraction (maximum amplitude 3.206±0.36N/g) but significantly reduced the relaxation induced by EFS. As shown in **figure 2**, L-NAME significantly suppressed nerve-mediated relaxation at all frequencies of stimulation above 2 Hz, suggesting that the major component of nerve-mediated GF relaxation was due to NO derived from NOS.

**Figure 3:**
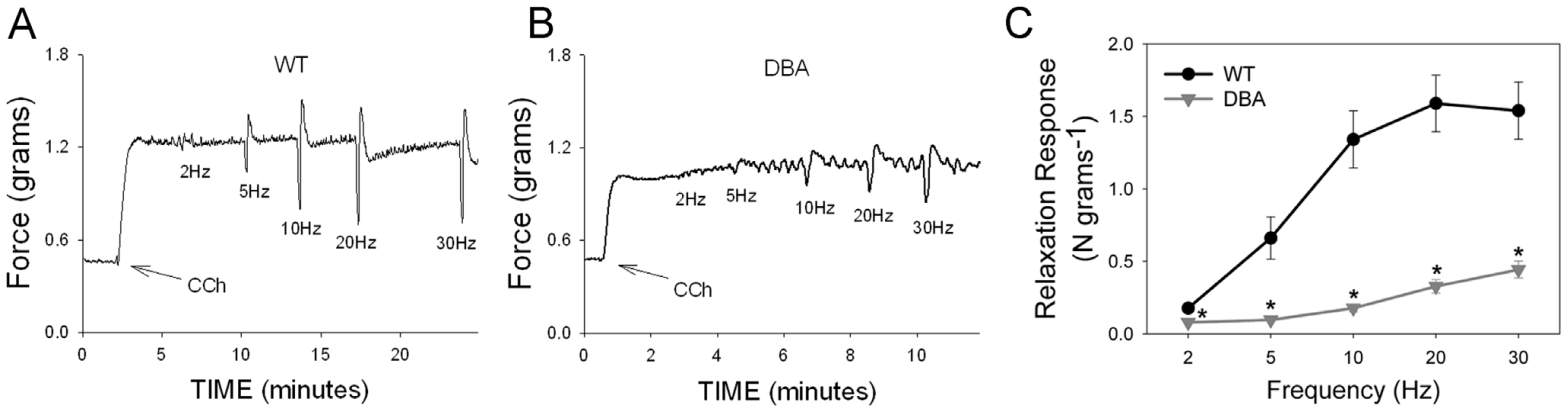
Effect of myosin Va deficiency on nerve mediated relaxation in mouse gastric fundus. (A) Representative tracing showing frequency dependent relaxation in a WT animal. (B) Representative tracing showing reduced relaxations in response to EFS in a DBA mouse. (C) Quantification of frequency-relaxation responses in WT and DBA mice. Note that in DBA mice, relaxation responses are markedly decreased. [n = 12 in each group; * = significantly lower than WT, unpaired t-test].

In the DBA mice, EFS also produced frequency dependent relaxation; however, this relaxation response was significantly lower than that in WT at all frequencies of stimulation (**figure 3**). Since the pre-contraction amplitude was significantly different between DBA and WT, the relaxation responses were normalized by the CCh pre-contraction in each tissue. EFS-induced relaxations were still significantly lower in DBA than in WT mice at all frequencies examined. At 20 Hz, the relaxation response in DBA mice was 16.1±1.8% of the pre-contraction which was significantly lower than the relaxation response (41.0±2.8%) in WT animals (p = 0.01, n = 12 in each group).

The relaxation response induced by EFS in the presence of L-NAME treatment in WT mice was compared with the nerve-mediated relaxation in DBA mice. The suppression of nerve mediated relaxation by L-NAME in WT mice at 20 Hz was not significantly different from the decreased relaxation response in DBA relative to WT (68.9±4.8% vs 67.3±6.5%). These observations suggest that nerve mediated nitrergic relaxation in GF was largely abolished in myosin Va deficient DBA mice.

**Figure 4:**
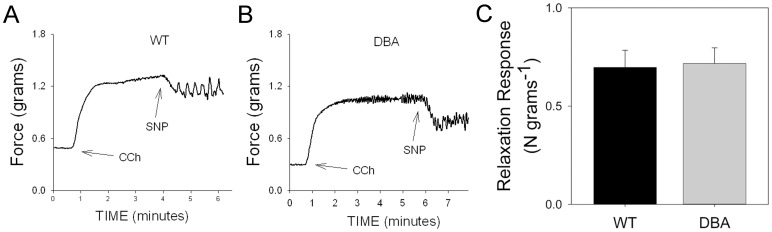
Effect of NO donor on contractile response. After pre-contraction with carbachol (arrows), relaxation was induced by SNP (1 µM) in WT (A) and DBA (B) fundus. (C) Graph shows that SNP induced relaxation was similar in magnitude in WT (black bars) and DBA (gray bars) mice. [n = 8 in each group, p = 0.433, unpaired t-test].

The relaxation responses in the fundus elicited by the addition of the NO donor SNP in CCh pre-contracted strips were not different between tissues of WT and DBA mice (**figure 4**), suggesting that myosin Va deficiency does not impair direct smooth muscle relaxation induced by NO.

### Nerve- mediated Relaxation in Corpora Cavernosa of Penis (CCP)

In penile cavernosal smooth muscle tissue, phenylephrine (PE) caused a stable, tonic contraction. The amplitude of contractile responses induced by PE was not significantly different between strains (0.45±0.09 N/g and 0.32±0.02 N/g respectively in WT and DBA).

**Figure 5:**
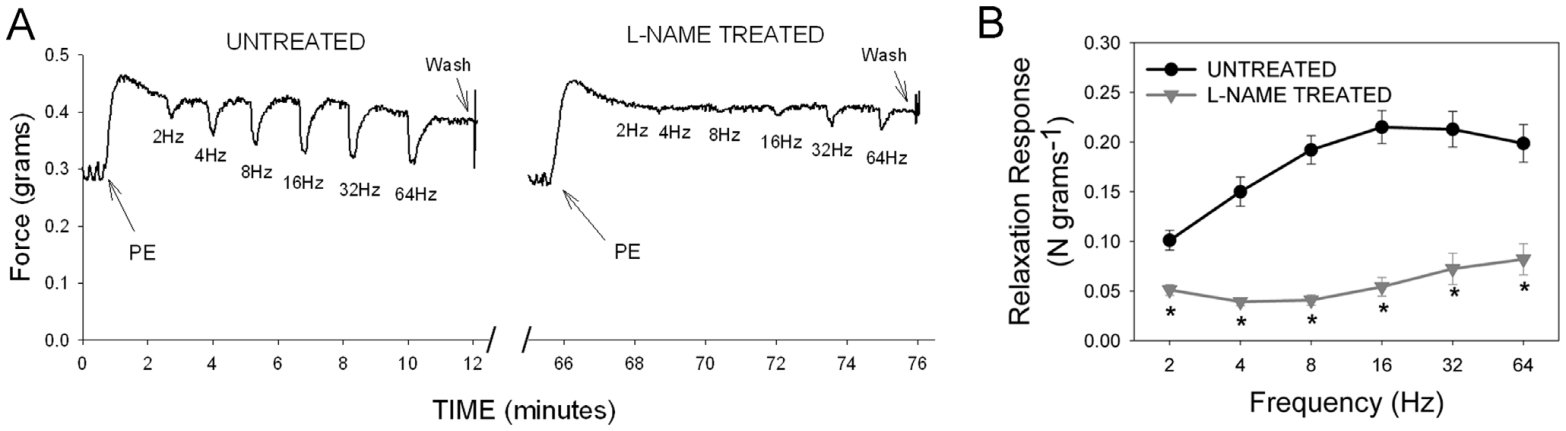
Effect of L-NAME on nerve-mediated relaxation in mouse corpora cavernosa of penis. (A) Representative tracing showing pre-contraction induced by phenylephrine (PE, arrows) and relaxation to increasing frequencies of stimulation in a WT mouse before (baseline) and after L-NAME treatment. Note the relaxation responses increased in amplitude with increasing stimulus frequencies. (C) In graph, frequency-relaxation responses under baseline conditions (black circles) were significantly attenuated by L-NAME (gray triangles) in WT mice. [n = 5; *significantly lower than baseline; p = 0.00002, paired t-test].

Pre-contracted CCP muscle strips from WT mice relaxed in response to EFS in a frequency dependent manner. Administration of L-NAME did not affect the amplitude of PE-induced pre-contraction in WT (0.33±0.05 N/g) but significantly reduced nerve-mediated relaxations at all the frequencies tested (**Figure 5**). The maximal suppression of relaxation by L-NAME at 16 Hz was 73.3±5.9% of the untreated response, consistent with CCP relaxation being predominantly mediated by NO derived from isoforms of NOS.

**Figure 6:**
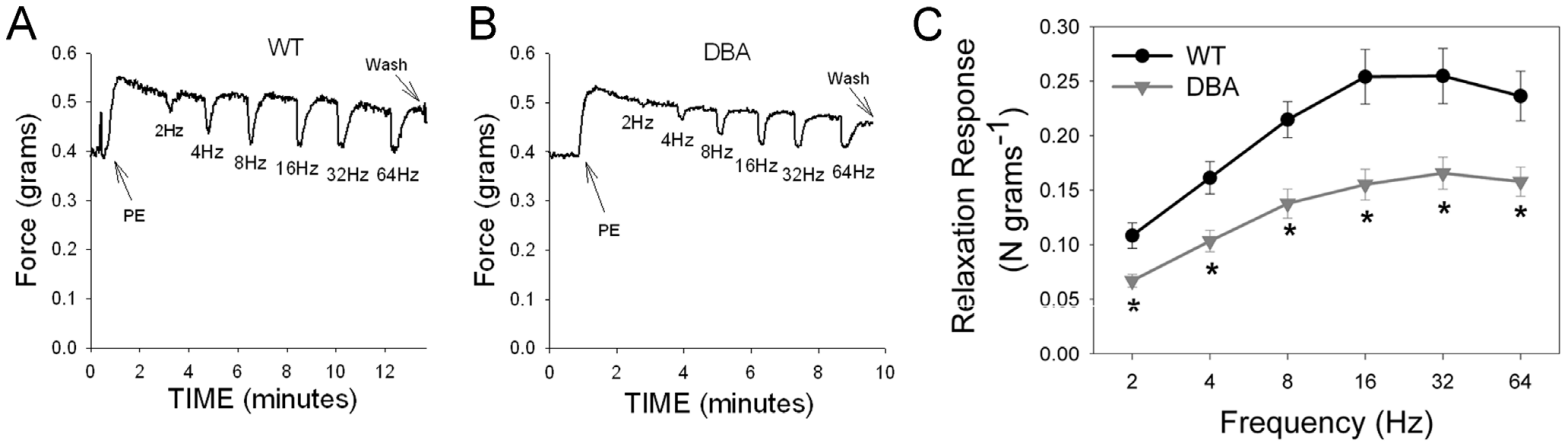
Effect of myosin Va deficiency on nerve mediated relaxation in mouse corpora cavernosa of penis. (A) Representative tracing showing frequency dependent relaxation of pre-contracted CCP (PE, arrow) in a WT animal. (B) Representative tracing showing relaxation responses induced by EFS in CCP of a DBA mouse. (C) Comparison of frequency-relaxation responses in WT (black circles) and DBA (gray triangles) CCP. [n = 12 for WT and n = 14 for DBA; * significantly lower than WT, unpaired t-test].

EFS-induced relaxation responses in DBA CCP tissue were significantly lower than those in WT CCP tissue at all frequencies (**Figure 6**). These observations suggest that nitrergic relaxation of the CCP is also dependent on myosin Va. Nerve stimulated CCP relaxation in DBA mice relative to WT was significantly greater (53.1±6.9%) than the L-NAME-resistant WT relaxation relative to the untreated response (25.2±4.4%, p<0.018, 16 Hz), indicating a residual NOS mediated relaxation component in DBA mice.

**Figure 7:**
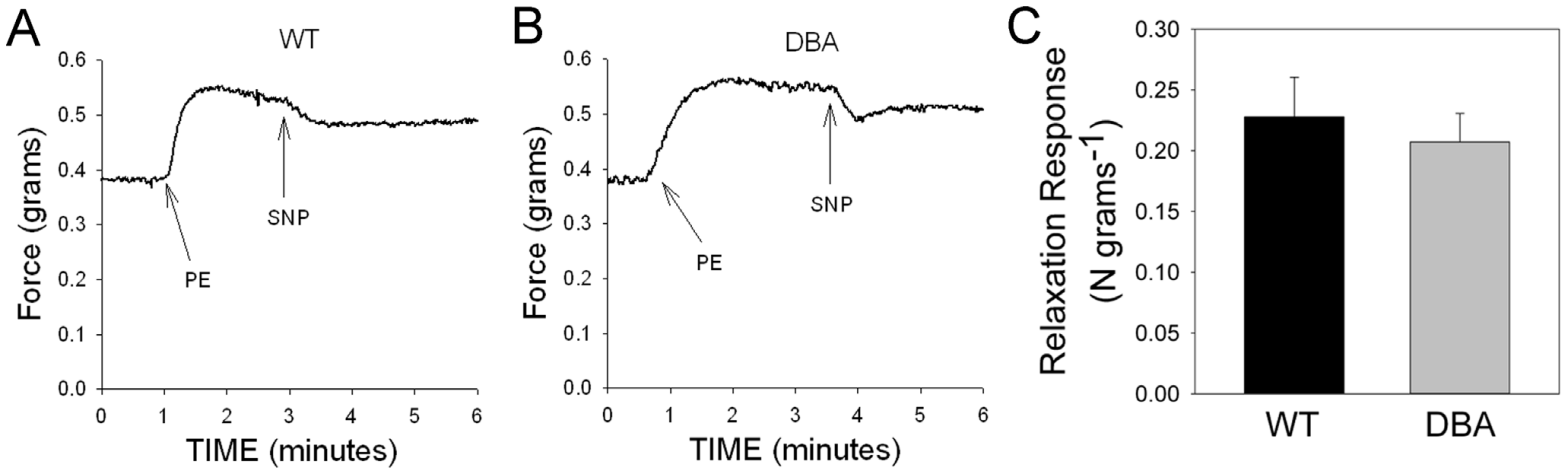
Effect of NO donor on contractile response in CCP. Representative tracing showing relaxation of cavernosal smooth muscle induced by SNP (1 µM) in WT (A) and DBA (B) mice after pre-contraction with phenylephrine (PE, arrow). (C) Graph compares the magnitude of SNP induced relaxation in WT (black circles) and DBA (gray triangles) mice. [n = 9 for WT and n = 10 for DBA cavernosal tissues, p = 0.302, unpaired t-test].

Cavernosal tissue from both WT and DBA mice, pre-contracted with PE, relaxed to the same degree in response to the NO donor SNP (1 µM) (**Figure 7**), suggesting that postjunctional smooth muscle responses to nitrergic relaxants were not modified by myosin Va deficiency in DBA mice.

### Comparison of Relaxation Responses in GF and CCP

To determine whether myosin Va differentially affected nitrergic relaxation in GF relative to CCP, we compared the NO-mediated relaxation response with the myosin Va-dependent relaxation response. The L-NAME sensitive relaxation response in WT mice relative to the untreated response was similar in GF compared to CCP (68.8±13% vs 74.8±4.4%, respectively; p = 0.44). The myosin Va dependent relaxation response, determined from the difference in relaxation between DBA and WT as a percent of the WT response, was significantly lower in CCP (38.9±5.8%) compared to GF (79.1±5.3%, p<0.001). Since the total NO mediated relaxation response in CCP was not different than in GF, this finding suggests that nitrergic relaxation may be more sensitive to loss of myosin Va in GF than in CCP.

## Discussion

The present study demonstrates an important role for myosin Va in nitrergic neurotransmission. We showed, using disparate smooth muscle neuromuscular junctions, that: 1) myosin Va localizes in nitrergic nerve fibers in GF and CCP; 2) nNOS interacts with myosin Va in both GF and CCP; 3) smooth muscle relaxation in response to nitrergic nerve stimulation is suppressed in DBA mice in both the GF and CCP due to impaired synthesis of NO rather than defective post-junctional NO signaling.

The loss of smooth muscle relaxation in response to neuronal stimulation in DBA mice is consistent with our previous electrophysiological studies showing loss of inhibitory junction potentials in these animals, despite normal hyperpolarization responses to exogenous NO donors [20]. In isolated enteric nerve varicosities from DBA animals, nNOSα expression was normal, but membrane associated nNOSα was decreased, and NO synthesis was impaired, showing that myosin Va deficiency may cause loss of catalytic nNOSα activity by preventing its membrane localization and thereby interfering with nitrergic neurotransmission [20]. The present mechanical study provides evidence for the functional importance of myosin Va in inhibitory neurotransmission and relaxation of GF smooth muscles. L-NAME, a nonselective NOS inhibitor suppressed neurally mediated relaxation by about 70%, indicating that the predominant component of nerve-mediated relaxation in GF was nitrergic in nature. The residual relaxation may be due to other inhibitory transmitters including ATP and VIP [26]–[27]. The magnitude of the relaxation response in DBA fundus was comparable to the L-NAME-resistant relaxation response in WT muscle strips, suggesting that myosin Va may be responsible for regulating nitrergic relaxation in GF. Further studies are needed to fully define the non-nitrergic and myosin Va insensitive inhibitory neurotransmitters in the stomach.

In the penis, neurogenic NO is fundamental to relaxation of cavernosal smooth muscle and initiation of erection [4], [6]. Accordingly, the present study showed that a dominant component of EFS induced CCP relaxation was mediated by NO released from penile nerves. Moreover, we showed that nitrergic relaxation of CCP was impaired in myosin Va deficient DBA mice, suggesting that nitrergic neurotransmission is regulated by myosin Va in the penis. Analogous to nNOSα trafficking in enteric nerve terminals, here we report the novel finding that transport of PnNOS to the varicosity membrane in penile nerves may be mediated by myosin Va. PnNOS differs from nNOSα by a 34 amino acid insert and is identical to nNOSµ expressed in skeletal and cardiac muscles [24], [28]. Nevertheless, several lines of evidence support the possibility of PnNOS cycling to the varicosity membrane in penile nerves. For example, PnNOS contains an N-terminal PDZ domain that can interact with the scaffold protein PSD95 (postsynaptic density 95 kDa) to facilitate its membrane localization [29]. In addition, the adapter protein LC8, which can bind nNOSα to myosin Va in enteric varicosities [20], is highly expressed in penile nerves and colocalizes with PnNOS [28]. We have shown here that myosin Va is localized to nNOS-positive nerve fibers in cavernosal tissue and interacts with nNOS. These observations suggest that myosin Va in penile nerves may act to transport PnNOS to the varicosity membrane for its catalytic activation and hence promote nitrergic neurotransmission in the penis.

The myosin Va dependent relaxation response induced by nerve stimulation was a smaller fraction of the WT response in CCP tissue compared to the relative responses in the GF. This difference was not due to a disparity in the overall extent of nitrergic relaxation in gastric and penile smooth muscle, as the nerve-mediated relaxation in the two tissues was similarly and effectively suppressed by a nonselective NOS inhibitor, L-NAME. These observations suggest that a portion of nerve-mediated nitrergic transmission in CCP may be independent of myosin Va. The larger residual nitrergic relaxation in CCP likely results from acetylcholine mediated eNOS activation [30]. In contrast to nitrergic relaxation in the GF, eNOS is an important source of NO in the CCP for cavernosal smooth muscle relaxation and sustained erection [4.6]. However, eNOS targeting to membrane caveolae [31] does not appear to require myosin Va. Resolution of the relative contribution of nNOS splice variants and eNOS, and role of myosin Va, require further studies.

The equivalent postjunctional responses of WT and DBA stomach and penis to exogenous NO suggest that the impaired nitrergic relaxation induced by EFS in DBA mice is due to defective synthesis of NO in nerve terminals, rather than a defect in NO signaling in smooth muscle. This deficit in NO synthesis may be related to inadequate localization of nNOS in the appropriate signaling compartment at the membrane that is required for its activation. Thus, the present study suggests that deficiency of the intracellular motor protein, myosin Va, may be involved in the pathogenesis of gastroparesis and erectile dysfunction.

In many disorders of gastrointestinal motility, impaired nitrergic function with near normal or only slightly reduced nNOS has been reported. For example, nNOS content was normal in 60 and 80% of gastric biopsies in idiopathic and diabetic gastroparesis, respectively [32]. Similarly, nNOS uncoupling has been shown to be responsible for dysfunctional nitrergic vasorelaxation of penile arteries from insulin-resistant obese Zucker rats [10]. Recently, expression of myosin Va has been reported to be reduced in neuronal tissues from the brain of animal models of diabetes mellitus [33]. Thus it is interesting to speculate that myosin Va defects may contribute to the pathogenesis of diabetic gastroparesis and erectile dysfunction.

If the function of nNOSα and pnNOS is regulated by myosin Va, the phenotype of DBA mice might be expected to resemble nNOS−/− mutants and other animal models of functional nNOS deficiency. DBA mice, an inbred strain of myosin Va deficiency, have a near normal life span, though they have been reported to be difficult breeders [34]. The *in vivo* phenotypes of the gut and penis in DBA mice are not well characterized. DBA mice have lower levels of physical activity and lower food intake than WT litters [35], but can present wide variation in food and water intake [36]–[37]. However, fecal output has not been properly quantified. The pathological changes in DBA mice were somewhat similar to those seen in nNOSα−/− mice. Mechanical studies have shown impaired nitrergic relaxation in nNOSα−/− mice [38], similar to that reported here for DBA mice. Although nNOS−/− mutants do not have breeding difficulty, few frank gastrointestinal symptoms have been reported, except gastric dilation which is dependent on the feeding behavior of the mutants [39]. Nevertheless, with careful testing, delayed gastric emptying, pyloric muscle thickening and motor dysfunction in nNOS−/− mutants were revealed. The presence of redundant pathways and compensatory mechanisms of relaxation may minimize the resultant pathology of nNOSα−/− mice. Likewise, the phenotype of DBA mice may be tempered by the hypomorphic character of the myosin Va deficiency. Further studies are needed to precisely define the functional phenotype of myosin Va deficiency.

In summary, our immunofluorescence and mechanical studies showed that myosin Va plays an important role in nitrergic neurotransmission in smooth muscle of the gastric fundus and penile corpora cavernosa as evidenced by the functional impairment of nitrergic relaxation demonstrated in an animal model of myosin Va deficiency. These studies suggest that loss of myosin Va contributes to functional abnormalities, including gastric motility disorders and erectile dysfunction.

## References

[1] Mashimo H, He XD, Huang PL, Fishman MC, Goyal RK (1996) Neuronal constitutive nitric oxide synthase is involved in murine enteric inhibitory neurotransmission. J Clin Invest 98: 8–13. 8690808 10.1172/JCI118781 PMC507393

[2] Toda N, Herman AG (2005) Gastrointestinal function regulation by nitrergic efferent nerves. Pharmacol Rev 57: 315–338. 16109838 10.1124/pr.57.3.4

[3] Toda N, Ayajiki K, Okamura T (2005) Nitric oxide and penile erectile function. Pharmacol Ther 106: 233–266. 15866322 10.1016/j.pharmthera.2004.11.011

[4] Dean RC, Lue TF (2005) Physiology of penile erection and pathophysiology of erectile dysfunction. Urol Clin North Am 32: 379–395. 16291031 10.1016/j.ucl.2005.08.007 PMC1351051

[5] Takahashi T (2003) Pathophysiological significance of neuronal nitric oxide synthase in the gastrointestinal tract. J Gastroenterol 38: 421–430. 12768383 10.1007/s00535-003-1094-y

[6] Andersson KE (2011) Mechanisms of penile erection and basis for pharmacological treatment of erectile dysfunction. Pharmacol Rev 63: 811–859. 21880989 10.1124/pr.111.004515

[7] Lefebvre RA (1995) Nitric oxide in the peripheral nervous system. Ann Med 27: 379–388. 7546628 10.3109/07853899509002591

[8] Alderton WK, Cooper CE, Knowles RG (2001) Nitric oxide synthases: structure, function and inhibition. Biochem J 357: 593–615. 11463332 10.1042/0264-6021:3570593 PMC1221991

[9] Gangula PR, Maner WL, Micci MA, Garfield RE, Pasricha PJ (2007) Diabetes induces sex-dependent changes in neuronal nitric oxide synthase dimerization and function in the rat gastric antrum. Am J Physiol Gastrointest Liver Physiol 292: G725–33. 17347455 10.1152/ajpgi.00406.2006 PMC2786258

[10] Sánchez A, Contreras C, Martínez MP, Climent B, Benedito S, et al (2012) Role of neural NO synthase (nNOS) uncoupling in the dysfunctional nitrergic vasorelaxation of penile arteries from insulin-resistant obese Zucker rats PLoS One. 7: e36027. 10.1371/journal.pone.0036027 PMC3335073 22540017

[11] Goyal RK, Chaudhury A (2010) Pathogenesis of achalasia: lessons from mutant mice. Gastroenterology 139: 1086–90. 20800108 10.1053/j.gastro.2010.08.013

[12] Mukhopadhyay S, Sekhar KR, Hale AB, Channon KM, Farrugia G, et al (2011) Loss of NRF2 impairs gastric nitrergic stimulation and function. Free Radic Biol Med 51: 619–25. 21605664 10.1016/j.freeradbiomed.2011.04.044 PMC3129370

[13] Villanueva C, Giulivi C (2010) Subcellular and cellular locations of nitric oxide synthase isoforms as determinants of health and disease. Free radical biology & medicine 49: 307–316. 20388537 10.1016/j.freeradbiomed.2010.04.004 PMC2900489

[14] Roszer T (2012) Harboring of NOS to the Cell Membrane. In: The Biology of Subcellular Nitric Oxide. Springer: 105–132.

[15] Chaudhury A, He XD, Goyal RK (2009) Role of PSD95 in membrane association and catalytic activity of nNOSαlpha in nitrergic varicosities in mice gut. Am J Physiol Gastrointest Liver Physiol 297: G806–813. Erratum in: Am J Physiol Gastrointest Liver Physiol (2010) 299: G100–2. 10.1152/ajpgi.00279.2009 PMC2763812 19679819

[16] Chaudhury A, Rao YM, Goyal RK (2008) PIN/LC8 is associated with cytosolic but not membrane-bound nNOS in the nitrergic varicosities of mice gut: implications for nitrergic neurotransmission. Am J Physiol Gastrointest Liver Physiol 295: G442–451. 18635601 10.1152/ajpgi.90280.2008 PMC2536782

[17] Magee TR, Ferrini MG, Davila HH, Zeller CB, Vernet D, et al (2003) Protein inhibitor of nitric oxide synthase (NOS) and the N-methyl-D-aspartate receptor are expressed in the rat and mouse penile nerves and colocalize with penile neuronal NOS. Biol Reprod 68: 478–488. 12533411 10.1095/biolreprod.102.007310

[18] Navarro-Lerida I, Martinez Moreno M, Roncal F, Gavilanes F, Albar JP, et al (2004) Proteomic identification of brain proteins that interact with dynein light chain LC8. Proteomics 4: 339–346. 14760703 10.1002/pmic.200300528

[19] Lajoix AD, Badiou S, Peraldi-Roux S, Chardes T, Dietz S, et al (2006) Protein inhibitor of neuronal nitric oxide synthase (PIN) is a new regulator of glucose-induced insulin secretion. Diabetes 55: 3279–3288. 17130471 10.2337/db06-0257

[20] Chaudhury A, He XD, Goyal RK (2011) Myosin Va plays a key role in nitrergic neurotransmission by transporting nNOSα to enteric varicosity membrane. Am J Physiol Gastrointest Liver Physiol 301: G498–507. 21680773 10.1152/ajpgi.00164.2011 PMC3174543

[21] Magee T, Fuentes AM, Garban H, Rajavashisth T, Marquez D, et al (1996) Cloning of a novel neuronal nitric oxide synthase expressed in penis and lower urinary tract. Biochem Biophys Res Commun 226: 145–151. 8806605 10.1006/bbrc.1996.1324

[22] Seperack PK, Mercer JA, Strobel MC, Copeland NG, Jenkins NA (1995) Retroviral sequences located within an intron of the dilute gene alter dilute expression in a tissue-specific manner. EMBO J 14: 2326–2332. 7774591 10.1002/j.1460-2075.1995.tb07227.x PMC398341

[23] Jenkins NA, Copeland NG, Taylor BA, Lee BK (1981) Dilute (d) coat colour mutation of DBA/2J mice is associated with the site of integration of an ecotropic MuLV genome. Nature 293: 370–374. 6268990 10.1038/293370a0

[24] Ignarro LJ, Bush PA, Buga GM, Wood KS, Fukuto JM, et al (1990) Nitric oxide and cyclic GMP formation upon electrical field stimulation cause relaxation of corpus cavernosum smooth muscle. Biochemical and biophysical communications 170: 843–50. 10.1016/0006-291x(90)92168-y 2166511

[25] Roder IV, Choi KR, Reischl M, Petersen Y, Diefenbacher ME, et al (2010) Myosin Va cooperates with PKA RIalpha to mediate maintenance of the endplate in vivo. Proc Natl Acad Sci U S A 107: 2031–2036. 20133847 10.1073/pnas.0914087107 PMC2836699

[26] Mulè F, Serio R (2003) NANC inhibitory neurotransmission in mouse isolated stomach: involvement of nitric oxide, ATP and vasoactive intestinal polypeptide. Br J Pharmacol 140: 431–7. 12970100 10.1038/sj.bjp.0705431 PMC1574027

[27] Gil V, Martínez-Cutillas M, Mañé N, Martín MT, Jiménez M, et al (2013) P2Y(1) knockout mice lack purinergic neuromuscular transmission in the antrum and cecum. Neurogastroenterol Motil 25: e170–82. 23323764 10.1111/nmo.12060

[28] Gonzalez-Cadavid NF, Burnett AL, Magee TR, Zeller CB, Vernet D, et al (2000) Expression of penile neuronal nitric oxide synthase variants in the rat and mouse penile nerves. Biol Reprod 63: 704–714. 10952911 10.1095/biolreprod63.3.704

[29] Brenman JE, Chao DS, Gee SH, McGee AW, Craven SE, et al (1996) Interaction of nitric oxide synthase with the postsynaptic density protein PSD-95 and alpha1-syntrophin mediated by PDZ domains. Cell 84: 757–767. 8625413 10.1016/s0092-8674(00)81053-3

[30] Kim N, Azadzoi KM, Goldstein I, Saenz de Tejada I (1991) A nitric oxide-like factor mediates nonadrenergic-noncholinergic neurogenic relaxation of penile corpus cavernosum smooth muscle. J Clin Invest 88: 112–8. 1647413 10.1172/JCI115266 PMC296010

[31] Musicki B, Burnett AL (2006) eNOS function and dysfunction in the penis. Experimental biology and Medicine 231: 154–165. 16446491 10.1177/153537020623100205

[32] Grover M, Farrugia G, Lurken MS, Bernard CE, Faussone-Pellegrini MS, et al (2011) NIDDK Gastroparesis Clinical Research Consortium. Cellular changes in diabetic and idiopathic gastroparesis. Gastroenterology 140: 1575–85. 21300066 10.1053/j.gastro.2011.01.046 PMC3081914

[33] da Costa AV, Calábria LK, Nascimento R, Carvalho WJ, Goulart LR, et al (2011) The streptozotocin-induced rat model of diabetes mellitus evidences significant reduction of myosin-Va expression in the brain. Metab Brain Dis 26: 247–51. 21842169 10.1007/s11011-011-9259-5

[34] McGill TE (1967) A double replication of a “small sample” study of the sexual behavior of DBA-2J male mice. Anat Rec 157: 151–153. 6040352 10.1002/ar.1091570205

[35] Jung AP, Curtis TS, Turner MJ, Lightfoot JT (2010) Physical activity and food consumption in high- and low-active inbred mouse strains. Med Sci Sports Exerc 42: 1826–33. 20216465 10.1249/MSS.0b013e3181daf5e8 PMC2935502

[36] Tsai PP, Stelzer HD, Hedrich HJ, Hackbarth H (2003) Are the effects of different enrichment designs on the physiology and behavior of DBA/2 mice consistent? Lab Anim 37: 314–327. 14599306 10.1258/002367703322389889

[37] Bachmanov AA, Reed DR, Beauchamp GK, Tordoff MG (2002) Food intake, water intake, and drinking spout side preference of 28 mouse strains. Behav Genet 32: 435–443. 12467341 10.1023/a:1020884312053 PMC1397713

[38] Kim CD, Goyal RK, Mashimo H (1999) Neuronal NOS provides nitrergic inhibitory neurotransmitter in mouse lower esophageal sphincter. Am J Physiol 277: G280–4. 10444441 10.1152/ajpgi.1999.277.2.G280

[39] Mashimo H, Kjellin A, Goyal RK (2000) Gastric stasis in neuronal nitric oxide synthase-deficient knockout mice. Gastroenterology 119: 766–73. 10982771 10.1053/gast.2000.16509

